# Etanercept/celecoxib on improving MRI inflammation of active ankylosing spondylitis: A multicenter, open-label, randomized clinical trial

**DOI:** 10.3389/fimmu.2022.967658

**Published:** 2022-08-26

**Authors:** Liudan Tu, Minjing Zhao, Xiaohong Wang, Qingcong Kong, Zena Chen, Qiujing Wei, Qiuxia Li, Qinghong Yu, Zhizhong Ye, Shuangyan Cao, Zhimin Lin, Zetao Liao, Qing Lv, Jun Qi, Ou Jin, Yunfeng Pan, Jieruo Gu

**Affiliations:** ^1^ Department of Rheumatology, Third Affiliated Hospital of Sun Yat-sen University, Guangzhou, China; ^2^ Department of Radiography, Third Affiliated Hospital of Sun Yat-sen University, Guangzhou, China; ^3^ Department of Rheumatology, ZhuJiang Hospital of Southern Medical University, Guangzhou, China; ^4^ Department of Rheumatology, Fourth People’s Hospital of Shenzhen City, Shenzhen, China

**Keywords:** spondyloarthritis, biological therapies, inflammation, NSAIDs, MRI

## Abstract

**Objective:**

To investigate the efficacy and safety of clinical, magnetic resonance imaging (MRI) changes in active ankylosing spondylitis (AS) patients with etanercept and celecoxib alone/combined treatment.

**Methods:**

A randomized controlled trial was conducted in three medical centers in China. Adult AS patients with BASDAI ≥4 or ASDAS ≥2.1, CRP >6 mg/L, or ESR 28 mm/1st hour were randomly assigned (1:1:1 ratio) to celecoxib 200 mg bid or etanercept 50 mg qw or combined therapy for 52 weeks. The primary outcomes were SPARCC change of the sacroiliac joint (SIJ) and spine and the proportion of patients achieving ASAS20 response at 52 weeks.

**Results:**

Between September 2014 and January 2016, we randomly assigned 150 patients (mean age, 32.4 years; mean disease duration, 109 months), and 133 (88.6%) completed the study. SPARCC inflammation scores of the SIJ and spine decreased in the three groups, and significant differences were found between the combined group and the celecoxib group [between-group difference: −6.33, 95% CI (−10.56, −2.10) for SIJ; −9.53, 95% CI (−13.73, −5.33) for spine] and between the etanercept group and the celecoxib group [between-group difference: −5.02, 95% CI (−9.29, −0.76) for SIJ; −5.80, 95% CI (−10.04, −1.57) for spine]. The ASAS20 response rates were 44%, 58%, and 84% in the celecoxib, etanercept, and combined groups, respectively, and a significant difference was only found between the combined and the celecoxib groups.

**Conclusion:**

Etanercept with or without celecoxib decreases inflammation detected by MRI at 1 year compared to celecoxib alone in active AS patients. The combination of etanercept and celecoxib was superior to celecoxib alone for the primary clinical response.

**Clinical Trial Registration:**

ClinicalTrials.gov, identifier NCT01934933.

## Introduction

Ankylosing spondylitis (AS) is a chronic inflammatory disease that mainly affects the sacroiliac (SI) joint and spine. New bone formation in the SI joint and spine, which may cause permanent impairment in spinal mobility and function, is an important pathological feature during disease development (1). Since AS peaks at the second to the third decade, Economic burden is huge from patient and society’s perspective (2). Therefore, treatments that can improve clinical symptoms, reduce inflammation, prevent structural damage, and maintain physical function are crucial.

Non-steroidal anti-inflammatory drugs (NSAIDs) are recommended as first-line therapy for AS, and a Cochrane study (3) showed that NSAIDs were efficacious in relieving pain and improving function among axial spondyloarthritis (axSpA) patients when compared with placebo. A single randomized control trial (RCT) also demonstrated that celecoxib might retard radiographic progression in AS patients with elevated C-reactive protein (CRP) (4). However, evidence of NSAIDs’ positive effect upon the Assessment of SpondyloArthritis International Society (ASAS) 20 response rate or MRI inflammation in the long term is limited. Tumor necrosis factor inhibitor (TNFi) was widely used in inflammatory diseases (5) and showed excellent ability in reducing pain and MRI inflammation (6, 7) when compared with placebo, with an ASAS20 response rate of 57%–78% after 12 or 24 weeks of treatment compared to 20%–30% in placebo (8, 9). TNFi and NSAIDs reduce pain and inflammation in different mechanisms, and whether combined TNFi and NSAID therapy has a superior effect on AS patients compared with solo treatment is still unknown. Although NSAIDs were applied in several RCTs comparing the efficacy of TNFi and placebo, the optional use of NSAIDs, of different categories and doses, was allowed.

So far, no clinical trial compares the effect of NSAIDs and TNFi alone/combined treatment on inflammatory change detected with MRI in active AS patients directly. The aim of this study is to compare the clinical and inflammatory change with etanercept and celecoxib alone/combined treatments in active AS patients over 52 weeks.

## Methods

### Trial design

This multicenter, open-label, randomized clinical trial was conducted at three centers in China from September 2014 to February 2016. Patients were screened by rheumatologists in clinic visits and assessed by researchers in the three centers. Ethics approval was reviewed and approved by the medical research ethics committee of three medical centers separately. The patients/participants provided their written informed consent to participate in this study.

### Participants

We included patients who met the 1984 modified New York criteria of AS (10), who had more than two and less than 16 syndesmophytes between the cervical and lumber spine detected by X-ray. Patients were included if their Bath Ankylosing Spondylitis Disease Activity Index (11) (BASDAI) ≥4 or Ankylosing Spondylitis Disease Activity Score (ASDAS) (12) ≥2.1, CRP >6 mg/L, or erythrocyte sedimentation rate (ESR) >28 mm/1st hour at baseline. The details of the inclusion and exclusion criteria are shown in the **Supplementary Appendix**.

### Randomization and masking

Participants in each site would be randomly assigned to etanercept or celecoxib or combined treatment in a ratio of 1:1:1 based on computer-generated random numbers. Allocation concealment was confirmed by a central automated distribution procedure that was independent of the investigators. This is an open-label study so that physicians and patients were aware of the allocated treatment groups after randomization. Follow-up assessments were done by trained research nurses who were masked to the allocated treatment groups.

### Interventions

Participants in the three groups were given one of the following three treatments: celecoxib 200 mg bid, etanercept 50 mg qw, and combined therapy for 52 weeks. During the study period, a certain concomitant treatment such as sulfasalazine or methotrexate with a stable dose (remains stable for 12 weeks prior to the study) would be allowed. NSAIDs (except celecoxib) and corticosteroids were prohibited during the study. NSAIDs should be stopped 7 days or five half-lives, and oral corticosteroids should be stopped 4 weeks prior to the administration of the study.

### Outcomes

The primary outcomes of this trial were changes in the Spondyloarthritis Research Consortium of Canada (SPRACC) scores of the SI joint (13) and spine (14) and ASAS20 response rate at week 52. The key secondary outcome measures were ASAS20 at other time points; ASAS40, 50, 70, 5/6 response rate; ASAS partial remission; BASDAI and ASDAS at each visit; the change of modified Stoke Ankylosing Spondylitis Spine Score (mSASSS); and SPRACC SI joint structural score from baseline to week 52. Additional physician-assessed secondary outcomes include physician global assessment of disease activity, Bath Ankylosing Spondylitis Metroloty Index (BASMI) (15), Maastricht Ankylosing Spondylitis Enthesitis Score (MASES), and tender joint count and swollen joint count at each visit. Patient-completed outcomes were visual analog scale scores (including patient global assessment of disease activity, pain, and back pain), AS-specific quality of life (ASQoL), and the Bath Ankylosing Spondylitis Functional Index (BASFI) at each visit. More details about the secondary outcomes appear in the Supplementary Appendix.

### MRI and radiographs

The MRI and radiographs of the SI joint and spine were assessed at baseline, 24 weeks, and 52 weeks. Scoring inflammation and structural damage in both the SI joint and spine are based on the SPARCC MRI scoring system (available on http://www.carearthritis.com/). mSASSS (16) was used to measure the quantification of chronic spinal changes by conventional spinal X-rays. More details about the MRI and radiographic scoring are seen in the Supplementary Appendix.

Two independent readers scored the images with masking to the treatment group, time sequence, and clinical data. The mean scores of the two readers were used for analysis unless a discrepancy exists. Discrepant situations included one reader considered the imaging to be unreadable, or if the scores moved in different directions (one positive, one negative), or differed by >5 points for SPARCC spinal and by >3 points for SPARCC SIJ (17) and by >5 points for the change of mSASSS (18). In those cases, a third reader (adjudicator) was required to assess the images, and the mean of the adjudicator’s score and the closest score of the two primary readers was considered as the final score. The two readers and one adjudicator were selected based on their experience with musculoskeletal image reading. The reliability of the SPARCC and mSASSS scores between readers was assessed using intraclass correlation coefficients (ICCs). The ICCs for all SI joint, spine SPARCC score, and mSASSS score were 0.82, 0.75, and 0.64, respectively. The ICCs for total change scores between time points were 0.71 for SIJ SPARCC scores, 0.74 for spine SPARCC scores, and 0.68 for mSASSS scores, respectively.

### Statistical analysis

According to a previous study evaluating the efficacy of adalimumab on decreasing spinal and SI joint inflammation of MRI (7), a median change of SPARCC spinal score of 6.3 (SD = 6) and 0.5 (SD = 1) in the adalimumab and placebo groups and a median change of SPARCC SI joint score of 3.6 (SD = 3) and 1.1 (SD = 1) in the adalimumab and placebo groups were reported. We calculated that a sample size of 20 evaluable patients per group (a total sample size of 60 participants) would provide 90% power to detect differences of spinal and SI SPRACC between the three treatment groups at the two-sided 5% significance level. For the co-primary outcome (ASAS20 at 52 weeks), under the assumption of a 70% response rate in the combined treatment group and 40% response rate in the celecoxib treatment group, it will provide at least 80% power to detect a 30% difference. To allow for a 20% dropout rate, a total of 150 patients (50 per group) were recruited.

Categorical variables were presented as counts and percentages. The Kruskal–Wallis rank test was used for comparison between groups using the full analysis set (all patients receiving ≥1 dose of the study drug). Non-responder imputation was used for missing ASAS20 responses. Continuous variables including the SPARCC score for the SI joint and spine were presented as means (SD) and analyzed as the change from baseline with a repeated-measure mixed model with terms for age, sex, body mass index, treatment, and trial center. The correlation within the repeated measures was addressed by using individual participant identification as a random effect. The effect of treatment was evaluated by the month × treatment interaction. Stata version 15.0 was used for all the analyses, and a *P*-value <0.05 (two-tailed) was regarded as statistically significant.

## Results

### Participants

**Figure 1:**
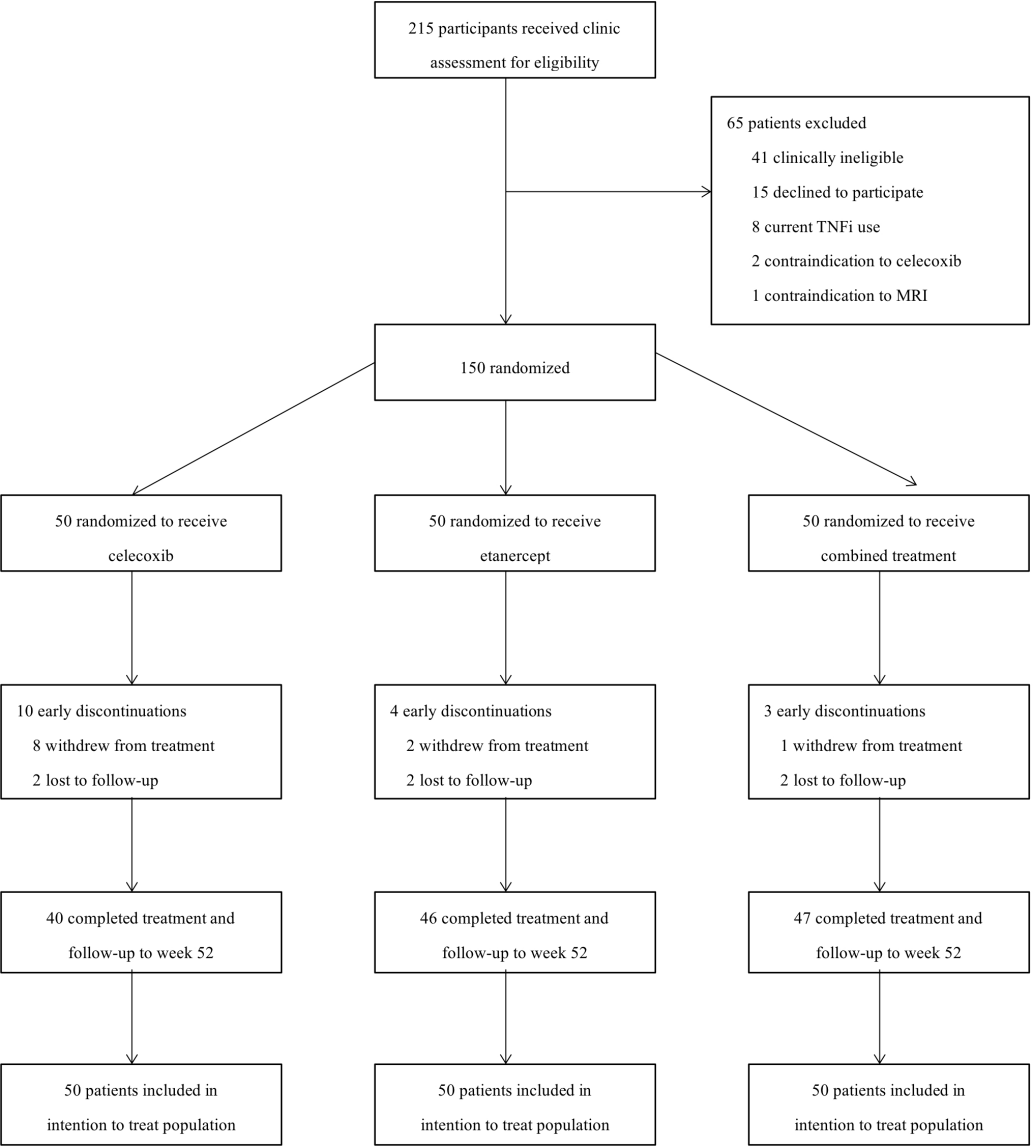
Study design and patient disposition.

**Figure 1** shows the flow of the study participants. A total of 215 participants were screened for eligibility from September 2014 to January 2016, and 150 (69.7%) were enrolled and randomly assigned (1:1:1) to one of the three treatment groups: celecoxib 200 mg bid (group A), etanercept 50 mg qw (group B), and combined therapy (group C) for 52 weeks. In group A, 40 (80%) of 50 patients completed the treatment and follow-up to week 52, and 46 (92%) and 47 (94%) did so in groups B and C, respectively. No significant difference was found between patients who completed and the dropouts (**Table S1**). No patients were found to be ineligible for the trial post-randomization. More patients (10 vs. 4 vs. 3) discontinued early from the trial in group A compared with the other two groups mainly due to lack of efficacy with celecoxib treatment.

The mean (SD) age of the participants in this study was 32.4 (8.37) years, 86% were men, and the mean disease duration was 109 (70) months. Patients included were in high disease activity with a mean BASDAI score of 5.2 (2.2) and a mean ASDAS-C score of 3.6 (0.87). Meanwhile, the mean syndesmophyte number detected by X-ray was 4.64 (3.22) and the mean mSASSS score was 12.12 (6.48). Participants’ demographic characteristics were comparable at baseline between the three groups (**Table 1**). Sixteen of 50 celecoxib-treated patients (32%), 17 of 50 etanercept-treated patients (34%), and 19 of 50 combined-treated patients (38%) had an SIJ SPARCC score of 0 at baseline, and no significant difference was found between the groups. All patients scored larger than 0 in the spinal SPARCC score at baseline.

**Table 1:** Baseline characteristics of AS patients in the three treatment groups.

	Celecoxib (*n* = 50)	Etanercept (*n* = 50)	Combined treatment (*n* = 50)
Sex (male), *n* (%)	38 (76%)	46 (92%)	45 (90%)
Age (years)	32.52 ± 8.22	33.12 ± 9.043	31.84 ± 7.896
Body mass index (kg/m^2^)	23.07 ± 3.762	22.07 ± 3.978	22.98 ± 3.836
HLA-B27 positive, *n* (%)	46 (92%)	48 (96%)	47 (94%)
Family history, *n* (%)	15 (30%)	14 (28%)	20 (40%)
Disease duration (months)	112.8 ± 81.73	106.7 ± 60.84	110.2 ± 66.46
BASDAI (0–10)	5.24 ± 1.895	5.425 ± 2.271	5.175 ± 2.319
BASFI (0–10)	2.9 ± 2.003	3.059 ± 2.329	3.383 ± 2.630
BASMI (0–10)	2 (1, 4)	1 (1, 4)	1 (1, 3)
MASES	0 (0, 2)	0 (0, 1)	0 (0, 1)
ASDAS-CRP	3.542 ± 0.747	3.621 ± 0.907	3.696 ± 0.928
ASDAS-ESR	3.171 ± 0.923	3.253 ± 1.005	3.289 ± 0.995
ASQoL	7.479 ± 4.708	8.180 ± 4.632	8.957 ± 5.324
ESR	24.5 (9, 38)	15.5 (11, 39)	20.5 (8, 37)
CRP	14.45 (9.1, 31.4)	14.7 (8.1, 24)	14.8 (7.8, 35.3)
Syndesmophytes	4.614 ± 2.738	4.844 ± 3.470	4.568 ± 3.295
mSASSS (0–36)	12.3 ± 6.219	12.58 ± 6.036	11.41 ± 7.34
SIJ SPARCC (0–72)	8.25 ± 11.56	8.279 ± 13.65	9.63 ± 13.08
Spine SPARCC (0–108)	24.51 ± 14.64	23.83 ± 10.70	27.32 ± 13.34
Structural change			
Erosion (0–40)	3.675 ± 5.868	3.780 ± 6.977	4.273 ± 7.951
Fat metaplasia (0–40)	8.175 ± 8.886	6.366 ± 8.581	7.432 ± 7.795
Backfill (0–20)	1.425 ± 2.669	1.146 ± 2.651	1.023 ± 2.774
Ankylosis (0–20)	5.45 ± 7.818	6.902 ± 8.634	4.409 ± 6.976

Values are mean (standard deviation) unless otherwise stated. One-way analysis of variance or the Kruskal–Wallis rank test was used for the comparisons between groups. HLA-B27, human leukocyte antigen-B27; BASDAI, Bath Ankylosing Spondylitis Disease Activity Index; BASFI, Bath Ankylosing Spondylitis Functional Index; BASMI, Bath Ankylosing Spondylitis Metroloty Index; MASES, Maastricht Ankylosing Spondylitis Enthesitis Score; ASDAS, Ankylosing Spondylitis Disease Activity Score; ASQoL, Ankylosing Spondylitis Quality of Life; ESR, erythrocyte sedimentation rate; CRP, C-reactive protein; mSASSS, modified Stoke Ankylosing Spondylitis Spine Score; SIJ, sacroiliac joint; SPARCC, Spondyloarthritis Research Consortium of Canada.

### SPARCC SIJ and spinal scores

**Figure 2:**
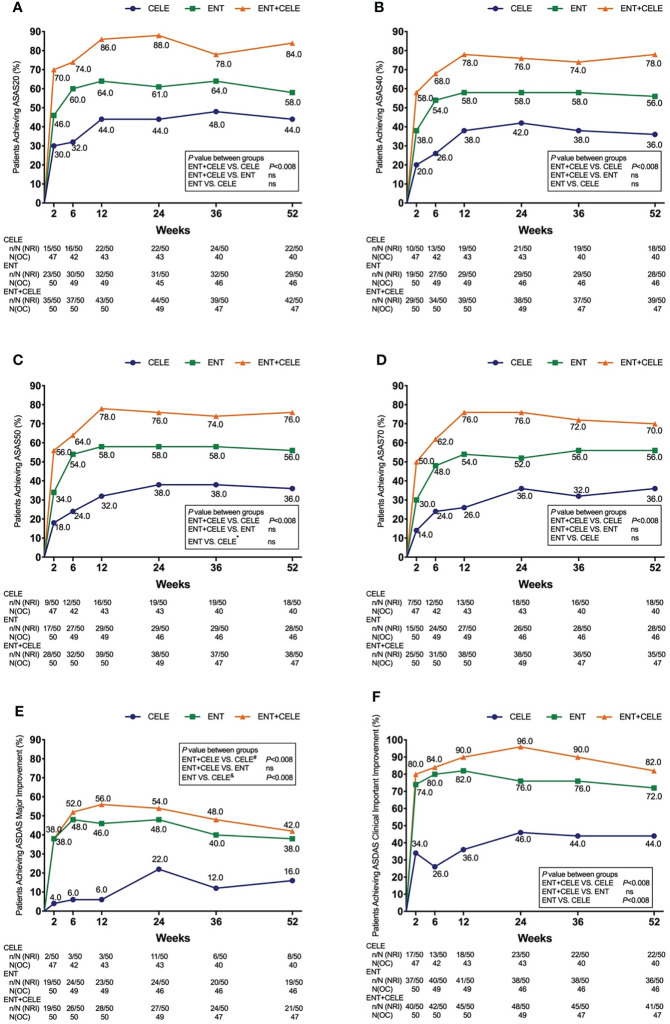
Mean change from baseline for **(A)** SPARCC MRI SIJ score and **(B)** SPARCC MRI spinal score. The mixed-effect model was used for the comparison between groups. *P < 0.05. Mean (SD) baseline values: **(A)** 8.63 (11.67) for CELE, 8.28 (13.65) for ENT, and 9.63 (13.08) for ENT+CELE; **(B)** 24.61 (14.05) for CELE, 24.14 (10.76) for ENT, and 27.80 (13.26) for ENT+CELE. Within-group *P*-value between baseline and week 52 from the mixed-effect model: <0.0001 for all in the SPARCC MRI SIJ and SPARCC MRI spinal scores. Within-group *P*-value between baseline and week 24 from the mixed-effect model: <0.0001 for all in the SPARCC MRI spinal score, <0.0001 for ENT and ENT+CELE in the SPARCC MRI SIJ score, and no significant difference for CELE in the SPARCC MRI SIJ score. ENT, etanercept; CELE, celecoxib; SIJ, sacroiliac joint; SPARCC, Spondylitis Research Consortium of Canada.

Changes in the SPARCC scores of the SI joint and spine are presented in **Table 2**. At baseline, the mean (SD) values of the SIJ and spinal SPARCC scores were 8.25 (11.5) and 24.51 (14.64) in group A, 8.28 (13.65) and 23.83 (10.70) in group B, and 9.63 (13.08) and 27.32 (13.34) in group C. The SIJ and spinal SPARCC scores decreased over 24 and 52 weeks in all the three groups, (**Figure 2**). At week 52, there were significant differences in the changes of the SIJ and spinal SPARCC scores between group C and group A and between group B and group A in the mixed-effect model. More improvement in the SIJ and spinal SPARCC scores was found in group C than in group B, but this difference did not reach statistical significance (**Table 2**). For the SIJ SPARCC score, a significant difference was found within group B [−7.44 (95% CI, −10.24 to −4.64), *P *< 0.001] and within group C [−8.75 (95% CI, −11.49 to −6.01), *P *< 0.001] from week 52 to baseline, while no significant difference was found within group A [−2.42 (95% CI, −5.64 to 0.80), *P* = 0.14] from week 52 to baseline. For the spinal SPARCC score, there were significant differences among the three groups from week 52 to baseline [group A: −13.71 (95% CI, −16.94 to −10.48), *P *< 0.001; group B: −19.51 (95% CI, −22.25 to −16.77), *P *< 0.001; group C: −23.23 (95% CI, −25.91to −20.56), *P *< 0.001]. At week 24, similar results were found in the changes of the SIJ SPARCC score between groups, while a significant difference was found between group C and group B in the spinal SPARCC score [between-group C–B difference, −4.06 (95% CI, −7.78 to −0.34), *P* = 0.03]. For within-group difference, the results of week 24 were similar to week 52 in both the SIJ and spinal SPARCC scores (**Figure 2**).

**Table 2:** Associations between treatment groups and changes in outcomes over 52 weeks.

	Group A	Group B	Group C	Between-group (C–A)^b^ difference in change, mean (95% CI), *P*-value	Between-group (C–B)^c^ difference in change, mean (95% CI), P value	Between-group (B-A)^d^ difference in change, mean (95% CI), *P*-value	*P* for trend
	Change^a^, mean (95% CI)	Change, mean (95% CI)	Change, mean (95% CI)				
SIJ SPARCC	−2.42 (−5.64, 0.80)	−7.44 (−10.24, −4.64)	−8.75 (−11.49, −6.01)	−6.33 (−10.56, −2.10), 0.003	−1.31 (−5.22, 2.61), 0.51	−5.02 (−9.29, −0.76), 0.02	0.005
Spinal SPARCC	−13.71 (−16.94, −10.48)	−19.51 (−22.25, −16.77)	−23.23 (−25.91, −20.56)	−9.53 (−13.73, −5.33), <0.001	−3.72 (−7.55, 0.11), 0.057	−5.80 (−10.04, −1.57), 0.007	<0.001
Structural change
Erosion	1.09 (0.15, 2.02)	−0.71 (−1.51, 0.10)	−0.86 (−1.64, −0.08)	−1.94 (−3.16, −0.72), 0.002	−0.15 (−1.27, 0.97), 0.79	−1.79 (−3.03, −0.55), 0.004	0.003
Fat metaplasia	−0.34 (−1.74, 1.06)	0.88 (−0.33, 2.09)	1.42 (0.25, 2.59)	1.76 (−0.06, 3.59), 0.06	0.55 (−1.14, 2.23), 0.53	1.22 (−0.63, 3.07), 0.19	0.06
Backfill	0.08 (−0.57, 0.72)	0.01 (−0.55, 0.58)	−0.15 (−0.69, −0.40)	−0.22 (−1.07, 0.62), 0.61	−0.16 (−0.94, 0.63), 0.69	−0.06 (−0.92, 0.79), 0.88	0.58
Ankylosis	0.72 (−0.10, 1.55)	0.11 (−0.60, 0.82)	0.70 (0.02, 1.39)	−0.02 (−1.09, 1.05), 0.97	0.59 (−0.39, 1.58), 0.24	−0.62 (−1.70, 0.47), 0.27	0.47
mSASSS	1.04 (0.15, 1.93)	0.95 (0.14, 1.76)	0.91 (0.10, 1.71)	−0.13 (−1.33, 1.06), 0.83	−0.05 (−1.19, 1.09), 0.93	−0.09 (−1.29, 1.11), 0.89	0.81
ASDAS-CRP	−1.21 (−1.43, −0.98)	−1.84 (−2.05, −1.63)	−2.05 (−2.26, −1.84)	−0.85 (−1.16, −0.54), <0.001	−0.21 (−0.51, 0.09), 0.16	−0.64 (−0.94, −0.33), <0.001	<0.001
BASDAI	−2.27 (−2.74, −1.79)	−3.03 (−3.47, −2.58)	−3.28 (−3.73, −2.84)	−1.02 (−1.66, −0.37), 0.002	−0.26 (−0.88, 0.36), 0.41	−0.76 (−1.40, −0.11), 0.02	0.002
BASFI	−0.73 (−1.18, −0.27)	−1.45 (−1.88, −1.02)	−2.05 (−2.47, −1.62)	−1.32 (−1.94, −0.69), <0.001	−0.59 (−1.19, 0.01), 0.053	−0.73 (−1.35, −0.10), 0.02	0.002
Back pain	−2.20 (−2.77, −1.62)	−3.30 (−3.84, −2.76)	−3.76 (−4.29, −3.22)	−1.56 (−2.35, −0.77), <0.001	−0.45 (−1.22, 0.30), 0.23	−1.10 (−1.89, −0.31), 0.006	<0.001
Patient global assessment	−1.59 (−2.15, −1.03)	−3.17 (−3.69, −2.64)	−3.73 (−4.25, −3.20)	−2.14 (−2.91, −1.38), <0.001	−0.56 (−1.30, 0.18), 0.14	−1.58 (−2.35, −0.81), <0.001	<0.001
CRP	−12.11 (−16.29, −7.92)	−13.25 (−17.17, −9.33)	−17.12 (−21.01, −13.23)	−5.02 (−10.73, 0.7), 0.08	−3.87 (−9.39, 1.65), 0.17	−1.14 (−6.88, 4.59), 0.69	0.08
ESR	−10.95 (−14.96, −6.94)	−14.10 (−17.86, −10.35)	−15.40 (−19.13, −11.68)	−4.45 (−9.92, 1.03), 0.11	−1.29 (−6.59, 3.98), 0.63	−3.15 (−8.64, 2.34), 0.26	0.11

Group A = celecoxib group; group B = etanercept group; group C = etanercept + celecoxib group. CI, confidence interval; SIJ, sacroiliac joint; SPARCC, Spondyloarthritis Research Consortium of Canada; mSASSS, modified Stoke Ankylosing Spondylitis Spine Score; ASDAS, Ankylosing Spondylitis Disease Activity Score; CRP, C-reactive protein; BASDAI, Bath Ankylosing Spondylitis Disease Activity Index; BASFI, Bath Ankylosing Spondylitis Functional Index; ESR, erythrocyte sedimentation rate.

a Changes in groups A/B/C are generated from the mixed models adjusted for age, sex, body mass index, and center, calculated using 52-week values minus baseline values.

b Between-group difference was calculated using group C values minus group A values.

c Between-group difference was calculated using group C values minus group B values.

d Between-group difference was calculated using group B values minus group A values.

### ASAS20 response rate

**Figure 3:**
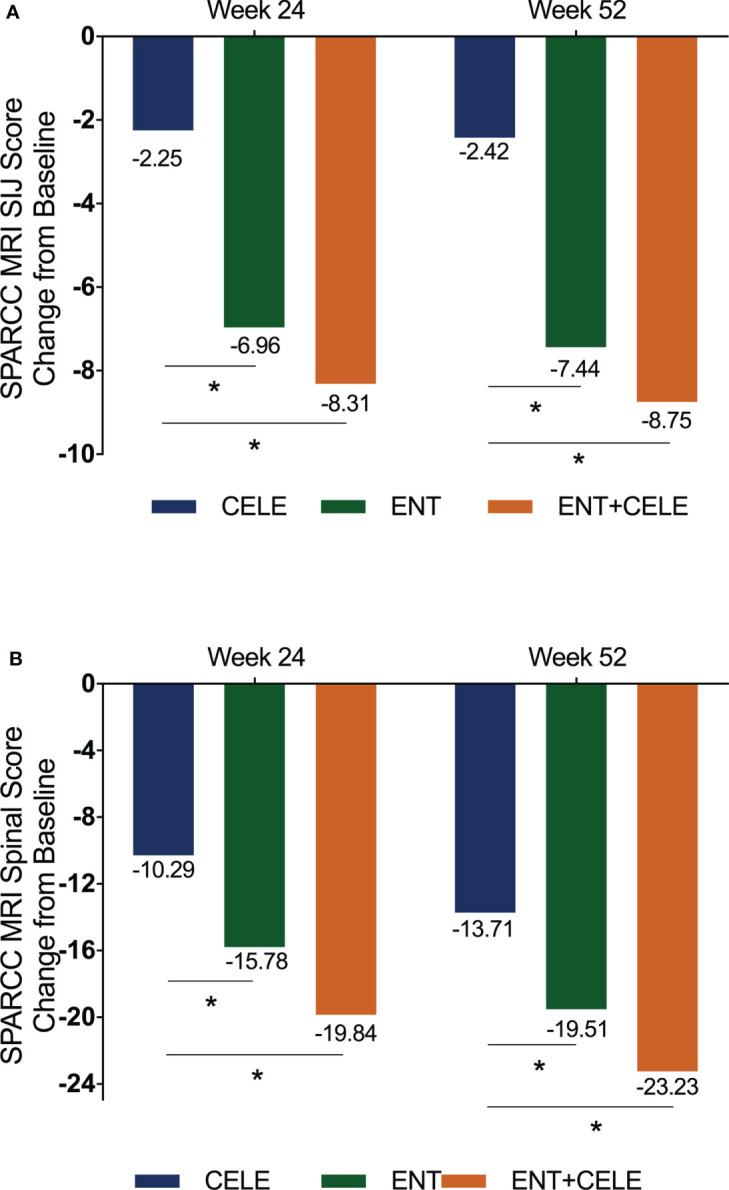
Proportion of patients achieving **(A)** ASAS20 response, **(B)** ASAS40 response, **(C)** ASAS50 response, **(D)** ASAS70 response, **(E)** ASDAS major improvement, and **(F)** ASDAS clinical important improvement in the three groups over 52 weeks. Population is modified intention to treat, non-responder imputation (NRI). The actual number of patients is shown as the observed case (OC). *P*-values for differences in the results between groups at any timepoint are from the Kruskal–Wallis test, and the adjusted *P*-value for significance is 0.008 in multiple comparisons between groups. ASAS, Assessment of SpondyloArthritis International Society; ASDAS, Ankylosing Spondylitis Disease Activity Score; ENT, etanercept; CELE, celecoxib; ns, non-significant. ^*^, significant difference was found between the etanercept and celecoxib groups only at week 2. ^#^, no significant difference was found between the etanercept + celecoxib and celecoxib groups at week 52. ^&^, significant differences were found between etanercept and celecoxib groups at weeks 2, 6, 12, and 36.

A total of 93/150 (51.28%) patients achieved ASAS20 using NRI by week 52.44% in group A, 58% in group B, and 84% in group C, and a significant difference was only found between group A and group C (*P* = 0.003) (**Figure 3**). Although no significant difference was found between group B and group C or between group B and group A, a slightly higher ASAS20 response rate was observed in group C than in group B at any time point.

### Other clinical efficacy outcomes

The proportions of all patients achieving ASAS40, ASAS50, ASAS70, ASAS5/6, and ASAS partial remission after 52 weeks of treatment were 56.7%, 56%, 54%, 52%, and 42.86%, respectively. At week 52, more patients in group C achieved an ASAS40, ASAS50, ASAS70, ASAS5/6 remission, and ASAS partial remission than in groups B and A (**Figure 3** and **Figure S1**), but a significant difference was only found between group C and group A.

More patients in groups C and B achieved ASDAS major improvement compared with group A over 52 weeks although a significant difference was observed at some time points (**Figure 3**). Over the course of treatment, groups C and B were significantly more effective than group A at achieving ASDAS clinical important improvement. For BASDAI, BASFI, ASDAS-C, and back pain, significant improvements were found between group C and group A and group B and group A (**Table 2**).

### Structural changes of the MRI and radiograph

The changes in the structural damages in the SIJ are presented in **Table 2**. Erosion decreased significantly in groups C and B after 52 weeks of treatment, and significant differences between group C and group A [between-group difference, −1.94 (95% CI, −3.16 to −0.72), *P* = 0.002] and between group B and group A [between-group difference, −1.79 (95% CI, −3.03 to −0.55), *P* = 0.004] were found. No significant difference was found between groups in terms of fat metaplasia, backfill, ankylosis, and mSASSS scores over 52 weeks (**Table 2**).

### Adverse events

Adverse events were reported in 55 (36.6%) of the 150 patients, and no significant difference was found between the three treatment groups (*P* = 0.09) (**Table 3**). No death occurred except for one serious adverse event that happened in this trial. One patient was admitted to the hospital because of drug dermatitis (not caused by etanercept). In all the 55 adverse events reported, the most commonly reported were infections, hepatobiliary disorders, and gastrointestinal upset. The AEs of the combined treatment were comparable with etanercept or celecoxib alone.

**Table 3:** Adverse events.

No. (%) of participants			
	Celecoxib (*n* = 50)	Etanercept (*n* = 50)	Combined treatment (*n* = 50)
Adverse events (total)	13 (26)	18 (36)	24 (48)
Infection			
Upper inspiration infection	4 (2.7)	7 (4.7)	9 (6)
Gastroenteritis	0	2 (1.3)	1 (0.7)
Urinary infection	2 (1.3)	1 (0.7)	0
Lymphnoditis	0	0	1 (0.7)
Gastrointestinal upset	4 (2.7)	0	4 (2.7)
Hepatobiliary disorders	1 (0.7)	4 (2.7)	6 (4)
Uveitis	1 (0.7)	0	1 (0.7)
Hypertension	0	1 (0.7)	1 (0.7)
Gout	0	1 (0.7)	0
Others^a^	1 (0.7)	1 (0.7)	1 (0.7)
Serious adverse events^b^	0	1 (0.7)	0

a Includes skin pruritus, hiccup, and insect bite.

b One patient was admitted to the hospital because of drug dermatitis (not caused by etanercept).

## Discussion

This study is the first to compare the SIJ and spinal SPARCC scores in active AS patients over 52 weeks in the three treatment groups consisting of celecoxib, etanercept, and combined treatment. The combination of etanercept and celecoxib was superior to celecoxib alone for the primary clinical response and MRI-based inflammation scores over 52 weeks. Etanercept with or without celecoxib decreases inflammation detected by MRI at 1 year compared to celecoxib alone.

MRI assessments provide an objective measure of detecting inflammation, and the SPARCC scoring system has been used widely and validated in AS patients (13, 14). TNFi has been known to reduce MRI inflammation in the SI joint and spine (6, 19), and improvement may be sustained 1 or 2 years or even longer (17, 20). Patients with higher BASDAI, CRP levels, and MRI inflammation are the most likely to respond to TNF blockers (21, 22). However, in most of the studies published before, MRI assessment of the SI joint and spine was not the primary endpoint of the original studies, and most of them compared TNFi with placebo. Even though the use of NSAIDs was allowed, the category and dose were not consistent. On the other hand, studies about the effect of NSAIDs on the inflammation of MRI in SpA/AS patients were limited (23, 24), and studies with long-term follow-up, appropriate control group, and larger sample size are needed. To our knowledge, this is the first study comparing NSAIDs with TNFi in long-term efficacy on MRI inflammation in active AS patients with an RCT design. We found that etanercept with or without celecoxib significantly improved SIJ and spinal SPARCC scores when compared with celecoxib over 52 weeks. No significant difference in the change of SIJ and spinal SPARCC scores was found when comparing combined treatment with etanercept alone. For celecoxib treatment, a significant difference in the change of SIJ SPARCC score was not found until week 52, which may indicate the slower process of celecoxib in reducing inflammation. The baseline SIJ or spinal SPARCC score was higher in our study, and it may be because the patients included in other studies were SpA patients with shorter disease duration (25, 26) or only BASDAI ≥4 was required (7).

Previous studies reported that the ASAS20 response rate varied from 29.5% (27) to 64.7% (28) after NSAID treatment in AS patients when compared with placebo in a relatively short term. TNFi was shown to be effective in achieving an even higher ASAS20 response rate when compared with placebo (8) with most of the patients using NSAIDs in both groups. However, no head-to-head comparison in ASAS20 response rate between TNFi and NSAIDs was found. Our study showed that the ASAS20 response rate was higher in groups C and B compared with that in group A (84% vs. 58% vs. 44%, *P *< 0.001) at 52 weeks, and a significant difference was only found between group C and group A (*P* = 0.003). The combination of celecoxib and etanercept treatment may be beneficial for sign and symptom remission with different mechanisms. Nevertheless, the possible complication of the long-term use of NSAIDs is an important concern that clinicians would advise to discontinue NSAID intake once symptoms improve or disappear with anti-TNF therapy. In a multicenter, randomized double-blind, placebo-controlled SPARSE study, the use of etanercept can significantly reduce NSAID intake (ASAS-NSAIDs score) compared with placebo over 8 weeks (29). The concept of combined TNFi and NSAID therapy may have an effect on retarding the radiographic progression of AS when inflammation is suppressed at the same time. While the protocol for a planned study comparing golimumab versus golimumab plus NSAID has been published, the results (assessing the retardation of radiographic progression in the spine) are not yet available (30). More well-designed studies with a long-term follow-up are needed to determine the potential structural benefits of NSAIDs and TNFi.

Prior studies of TNFi in AS have generally employed plain radiographs of the spine to assess radiographic progression that requires long periods of follow-up and large numbers of study subjects due to slow rates of damage accumulation on radiographs. In contrast, we assessed structural lesions using MRI. More attention was paid to structural lesions detected with the T1 sequence of MRI in recent years. Erosion on SIJ MRI was reported to be a highly specific lesion in patients with SpA (31) and to enhance the diagnostic utility in early SpA (32). Erosion may occur in SpA without radiographic change and even in the absence of SIJ bone marrow edema on MRI (33). A study demonstrated that etanercept was associated with a significantly greater reduction in erosion and an increase in backfill at 12 weeks compared with placebo (34), while no difference was observed between adalimumab and placebo for structural lesions (35). In this study, erosion was found to be decreased more in the combined as well as in the etanercept treatment when compared with celecoxib treatment, while no significant difference was found in the other structural lesions like fat metaplasia, backfill, and ankylosis. The possible reason may be that the morphology of erosion may change as the inflammation resolves and the evolution of erosion to fat metaplasia or backfill may take time following inflammation resolution.

The limitations of this trial include the open-label design, which might have caused unintentional bias in favor of the etanercept or combined treatment group, and even though we used masked nurses to conduct the assessments, this problem may still be existing. The structural changes of the radiograph, with a minimum interval time of 2 years, were not designed to be the primary outcome, so only *post-hoc* analysis can be conducted in this study.

In summary, the results of this trial demonstrate that etanercept with or without celecoxib is clinically effective and has an excellent anti-inflammatory effect assessed by MRI over 52 weeks in patients with active AS compared with celecoxib treatment. This novel study, which allows for a direct comparison of NSAIDs and TNFi on active AS patients, provides clear evidence for the efficiency of anti-inflammatory in the three treatment options.

## Data availability statement

The original contributions presented in the study are included in the article/**Supplementary Material**. Further inquiries can be directed to the corresponding author.

## Ethics statement

This study was reviewed and approved by the Third Affiliated Hospital of Sun Yat-sen University, ZhuJiang Hospital of Southern Medical University and Fourth People's Hospital of Shenzhen City. The patients/ participants provided their written informed consent to participate in this study.

## Author contributions

JG had full access to all of the data in the study and takes responsibility for the integrity of the data and the accuracy of the data analysis. Study concept and design: JG, LT, MZ, QY, and ZY. Acquisition, analysis, or interpretation of data: all authors. Drafting the manuscript: LT, MZ, and JG. Critical revision of the manuscript for important intellectual content: all authors. Statistical analysis: LT, ZC, and JG. All authors contributed to the article and approved the submitted version.

## Funding

This study was funded by Pfizer Ltd. The funder was not involved in the study design, collection, analysis, interpretation of data, the writing of this article, or the decision to submit it for publication.

## Acknowledgments

We especially thank the participants who made this study possible, and we gratefully acknowledge the role of the study staff and volunteers in collecting the data.

## Conflict of interest

The authors declare that the research was conducted in the absence of any commercial or financial relationships that could be construed as a potential conflict of interest.

## Publisher’s note

All claims expressed in this article are solely those of the authors and do not necessarily represent those of their affiliated organizations, or those of the publisher, the editors and the reviewers. Any product that may be evaluated in this article, or claim that may be made by its manufacturer, is not guaranteed or endorsed by the publisher.
