# Supplementary material for: Etanercept/celecoxib on improving MRI inflammation of active ankylosing spondylitis: A multicenter, open-label, randomized clinical trial
Source: Front Immunol. 2022 Aug 26;13:967658. doi: 10.3389/fimmu.2022.967658 (PMC9458864; doi:10.3389/fimmu.2022.967658)
Supplement: Supplementary file 1 [file DataSheet_1.docx]

Supplementary appendix

**Title**

Etanercept/celecoxib on improving MRI inflammation of active ankylosing spondylitis：a multi-center, open label, randomized clinical trial

**Sponsor**

This study was funded by Pfizer Ltd.

**Contact details**

Prof Jieruo Gu

Department of Rheumatology, Third Affiliated Hospital of Sun Yat-sen University

Guangzhou, Guangdong, China, 510630

Telephone numbers: +8613922280820

Trial Protocol

1.Introduction

A number of studies have demonstrated the efficacy of tumor necrosis factor-α inhibitors (TNFi) in reducing inflammation of Sacroiliac Joints (SIJ) and Spine in ankylosing spondylitis (AS) patients. However, the effect of TNFi on retarding radiographic progression in AS patients is still controversial. Interestingly, one clinical trial reported that a strategy of continuous use of NSAIDs (celecoxib) reduced radiographic progression in symptomatic patients with AS. The underlying mechanism is still unknown. It is possible that while the TNFi control inflammation very effectively, it does not have much effect on retarding new bone formation. The NSAIDs on the other hand may not be as effective in controlling inflammation but do inhibit the pathogenic processes involved in bone changes that occur in AS patients. So AS patients may have a better outcome (both control of inflammation and structural changes) if treated with a combination of NSAID and TNFi. This study is designed to assess the clinical and structural outcomes in AS patients treated with a combination of an NSAID (celecoxib) + TNFi (etanercept) versus celecoxib or etanercept alone.

2.Study objective and outcomes

2.1 Objectives

This study is designed to evaluate the effect of celecoxib and/or etanercept treatment on clinical and structural changes in active AS patients after 52 weeks as measured by clinical, MRI and radiographic assessments. The structural changes seen by MRI and radiographic measures will also be compared and correlated. The safety information will also be collected and analyzed during the study. The safety information includes adverse event (AE), serious adverse event (SAE) and clinically significant abnormal laboratory tests.

2.2 Outcomes

There are two primary outcomes in this study. The first one is to compare the Spondyloarthritis Research Consortium of Canada (SPARCC) score of SIJ and spine from baseline to week 52 in three treatment groups in active AS patients. The co-primary outcome is to compare the Assessment of Spondyloarthritis International Society (ASAS) 20 response rate of three treatment groups at week 52.

Secondary outcomes include ASAS40, ASAS70, ASAS5/6 response rate, ASAS partial remission, Ankylosing Spondylitis Disease Activity Score (ASDAS) remission and ASDAS low disease activity, Bath Ankylosing Spondylitis Disease Activity Index (BASDAI), Bath Ankylosing Spondylitis Functional Index (BASFI), Bath Ankylosing Spondylitis Metroloty Index (BASMI), Masstricht Ankylosing Spondylitis Enthesitis Score (MASES) and safety outcomes.

3.Study design

This is a prospective, open label, active controlled, parallel group study. A total of 150 active AS patients will be randomized in a 1:1:1 ratio into the following three treatment groups: celecoxib 200mg twice a day, etanercept 50mg weekly, celecoxib 200mg twice a day plus etanercept 50mg weekly for 52 weeks. This study will be registered in the Clinical Trials.

4.Subject Selection

4.1 Inclusion Criteria

Patients are eligible to be included in the study only if they meet all of the following criteria at screening or as specified:

[1] Subject is 18-65 years old at the screening age.

[2] Subject meets 1984 modified New York criteria of ankylosing spondylitis (AS) diagnosis (have unilateral sacroiliitis grade 3-4 or bilateral sacroiliitis grade 2-4, plus at least one of the following three clinical criteria: a. low back pain last more than 3 months, improved with exercise but not with rest. b. limitation of lumbar spine movement in sagittal and frontal planes. c. chest expansion decreased relative to normal values for age and sex.).

[3] Subject has active AS defined as the following three aspects: BASDAI≥4 or ASDAS score ≥2.1; CRP>6mg/L or ESR >28mm/1^st^ hour;

[4] Subject has sydesmophytes ≥2 and <16 measured by X-ray from cervical to lumbar spine;

[5] Fertile women must agree and commit to use a medically accepted form of contraception during the study and for at least 12 weeks after the last dose of test article;

[6] No evidence of active or latent or inadequately treated infection with Mycobacterium tuberculosis.

4.2 Exclusion Criteria

[1] Pregnant women.

[2] Subject has a history of infection requiring hospitalization and parenteral anti-microbial, or otherwise judged clinically significant by the investigator within the 24 weeks prior to screening.

[3] Subject is receiving TB chemoprophylaxis during screening and has had ALT and /or AST >2-times the upper limit of normal during this period.

[4] Subject has received a TNFi or other biologics within 12 weeks prior to screening.

[5] Subject has parenteral or intra-articular injections of corticosteroids within 12 weeks prior to screening.

[6] Subject has psoriasis or IBD.

[7] Subject has diabetes with uncontrolled hypoglycemia.

[8] Subject has a history of hypersensitivity to any of the study medications and sulfonamides or components of the concomitant medicines.

[9] Subject has a history of asthma, urticarial, or other allergic-type reactions after taking aspirin or other NSAIDs.

[10] Subject has a history of cancer or lymphoproliferative disease.

[11] Subject has a history of psychiatric disease, intellectual deficiency, poor motivation or other conditions that will limit the validity of informed consent to participate in the study or the potential compliance to study procedures.

[12] Subject has a history of alcohol or drug abuse that would interfere with the subject’s ability to comply with protocol requirements.

[13] Subject currently or recently has a history of uncontrolled clinically significant disease including gastrointestinal ulcer, active gastrointestinal bleeding, myocardial infarction within 12 months, unstable angina pectoris, congestive heart failure, cardiac surgery and the perioperative period in the setting of coronary artery bypass graft surgery.

[14] Study investigators, sub-investigators, study coordinators, employees of a participating investigator or immediate family members of the aforementioned are excluded from participating in this study.

5.Study treatments

5.1 Allocation to treatment

Patient eligibility will be determined at the screening and baseline visits, at which time the patient will be randomized (defined as the process of receiving medication and randomization number from SPSS v 17.0).

5.2 Preparation and Dispensing

Injections should never be administered consecutively in the same location. Instead, alternate sites (left and right arms, left and right thighs, left and right sides of the abdomen) should be used with each administration. New injections should be given at least 1 inch from the old sites and never into areas where the skin is tender, bruised, red, or hard.

5.3 Concomitant medication

During the study a stable dose of sulfasalazine or methotrexate for AS will be allowed. Doses of such concomitant medications should remain stable 12 weeks prior to study and during the study. NSAIDs (except celecoxib) and corticosteroids are prohibited during the study. NSAIDs should be stopped 7 days or 5 half-lives (whichever is longer); oral corticosteroids should be stopped 4 weeks prior to administration of study drugs. Aspirin 75 mg per day for cardiovascular protection is allowed. Proton pump inhibitors (PPIs) should be considered for patients treated with celecoxib and with GI risk. Concomitant medication can be stopped or adjust dosage for safety reason in the discretion of investigator. All adverse events related with the concomitant medicine should be recorded.

6.Study procedures

Timetable and measures to be made

|  | screen | Week 0 | Week 2 | Week 6 | Week 12 | Week 24 | Week 36 | Week 52 | Early end of treatment |
| --- | --- | --- | --- | --- | --- | --- | --- | --- | --- |
| Informed consent | × |  |  |  |  |  |  |  |  |
| Specific medical history | × |  |  |  |  |  |  |  |  |
| General medical history | × |  |  |  |  |  |  |  |  |
| Focused history and physical examination | × | × | × | × | × | × | × | × | × |
| Weight | × | × | × | × | × | × | × | × | × |
| Laboratory^a^ | × | × | × | × | × | × | × | × | × |
| ECG | × | × | × | × | × | × | × | × | × |
| Registration | × |  |  |  |  |  |  |  |  |
| Randomization |  | × |  |  |  |  |  |  |  |
| Chest X-ray | × |  |  |  |  | × |  | × | × |
| X ray sacroiliac and whole spine | × |  |  |  |  | × |  | × | × |
| MRI sacroiliac joint & spine | × |  |  |  |  | × |  | × | × |
| Assessments |  |  |  |  |  |  |  |  |  |
| Efficacy | × | × | × | × | × | × | × | × | × |
| Safety | × | × | × | × | × | × | × | × | × |
| Concomitant medication |  |  |  |  |  |  |  |  |  |
| Biomarker analysis^b^ |  | × |  |  | × | × |  | × |  |

^a^ laboratory: hematology, blood chemistry, urinalysis, coagulation, ESR, CRP are required at all visits. Pregnancy test is required in female at childbearing age at screening.

^b^ Biomarker analysis: blood sample will be collected in patients who sign separate informed consent form for later biomarker and genomic analysis.

7.Assessments

7.1 Clinical Response Assessment

Clinical efficacy endpoint assessments include ASAS20, ASAS40, ASAS50, ASAS70, ASAS5/6, ASAS partial remission, BASDAI, BASFI, ASDAS, number of tender joints, number of swollen joints, MASES, BASMI, dactylitis, physician and patient global assessments.

The ASAS20, ASAS50, ASAS40 and ASAS70 are composite measures derived from four patient domains such as the patient global assessment of disease activity, spinal pain, function (Bath Ankylosing Spondylitis Functional Index [BASFI]) and inflammation (mean of BASDAI question #5 [intensity of morning stiffness] and #6 [duration of morning stiffness]). An ASAS20 response is defined as at least 20% improvement and an absolute improvement from baseline of at least 1 unit (0-10) in the remaining domain. An ASAS40 response is defined as at least 40% improvement and an absolute improvement from baseline of at least 2 units (0-10) in at least three of the four domains without any worsening in the remaining domain. An ASAS50 response is defined as at least 50% improvement and an absolute improvement from baseline of at least 2 units (0-10) in at least three of the four domains without any worsening in the remaining domain. An ASAS70 response is defined as at least 70% improvement or an absolute improvement from baseline of at least 2 units (0-10) in at least three of the four domains without any worsening in the remaining domain. ASAS partial remission was defined as a value below 2 on a scale of 0 to 10 in each of the 4 ASAS domains. An ASAS5/6 response was defined as at least 20% improvement in five out of six ASAS domains, plus spinal mobility and C reactive protein.

The BASDAI is a six-item questionnaire used to assess the severity of five major symptoms of AS, including fatigue, spinal pain, peripheral arthritis, enthesitis, intensity of morning stiffness, and duration of morning stiffness using a 0-10 VAS scale. The Ankylosing Spondylitis Disease Activity Score (ASDAS) is used to assess disease activity in AS patients in five domains: total back pain (BASDAI item 2), patient global assessment, peripheral pain, and swelling (BASDAI item 3), duration of morning stiffness (BASDAI item 6), and CRP (mg/L). Four disease activity states were divided according to the ASDAS score cut-offs: <1.3 “inactive disease”, 1.3-2.1 “low disease activity”, 2.1-3.5 “moderate disease activity”, >3.5 “high disease activity”. For improvement scores, a major improvement (ASDAS MI) was defined as a decrease from baseline ≥2.0, and a clinically important improvement (ASDAS CII) was defined as a change ≥1.1 from baseline.

7.2 Safety Assessment

Collect and record AE anytime it occurs. Physical examination, vital sign measurement and laboratory tests such as hematology, urinalogy, blood biochemistry test will be performed.

7.3 MRI assessment

MRI was performed with 1.5 Tesla (Siemens, Erlangen, Germany) systems using propriate suface coils. Sequences including T1-weighted spin echo (T1WSE) and short-tau inversion recovery (STIR) were acquired in a coronal plane titled parallel to the long axis of the SJ joint with 3-4 mm slice thickness and 12-15 slices acquired.

SPARCC of SIJ and spine

SPARCC SIJ scores were based on the measurement of six consecutive slices, with largest score of 12 for edema, intensity and depth per slice and a total score of 0-72. Three consecutive slices of whole spine were measured and six most severely affected discovertebral units per slice were chosen to give a maximal score of 6 for edema, intensity and depth in one discovertebral unit for SPARCC spinal score. A total of 0-108 score was calculated and with a higher score indicating greater inflammation.

SPARCC MRI Sacroiliac Joint Structural Score

SPARCC SSS was used to assess the structure lesions basing on T1WSE sequences. Four kinds of lesions are assessed based on five consecutive slices through the SIJ. Fat metaplasia is defined as an increased signal in bone marrow on T1WSE and the lesion has to demonstrate homogeneous signal with more than 1 cm in depth from the joint surface. Erosion is defined as the full-thickness loss of the dark appearance of either iliac or sacral cortical bone at its anticipated location and loss of the normal bright appearance of adjacent bone marrow. Backfill is defined as complete loss of iliac or sacral cortical bone at its anticipated location and increased signal that is clearly demarcated from adjacent normal marrow by irregular dark signal reflecting sclerosis at the border of the eroded bone. Ankylosis is defined as bone marrow signal on T1WSE sequences extending between the sacral and iliac bone marrow with a full-thickness loss of the dark appearance of the iliac and sacral cortical bone. The presence/absence of lesions is scored using an online data entry system in SIJ quadrants (fat, erosion) or halves (backfill, ankylosis) with a scoring range of 0-40 for quadrants lesions and 0-20 for halves lesions.

7.4 Radiographic assessments

The modified Stroke Ankylosing Spondylitis Score (mSASSS) was used to assess the structural change of AS patients. It assesses the anterior part of the cervical and lumbar spine (from the lower corner of T12 to the upper corner of S1 and from the lower corner of C2 to the upper cornor of T1) as follows: 0= normal; 1= erosion, sclerosis or squaring; 2= syndesmophytes; 3=bridging syndesmophytes (the total score ranges from 0 to 72).

8.Subject Withdrawal

Once a subject is randomized to investigational product, the subject’s vital status will be tracked for the duration of the study. Subjects may choose to discontinue use of investigational product at any time but full accountability at the end of the study is required of all subjects.

If a subject withdraws or discontinued for any reasons prior to last visit, e.g. to withdraw IFC, early withdraw from the study due to AE etc., an early end of treatment assessments listed in ”Early end of treatment” of the study flow chart at the last visit must be completed.

8.1 Withdrawal from Investigational Product

A subject may voluntarily discontinue investigational product at any time. The investigator may also, at his or her discretion, discontinue a subject from receiving investigational product at any time. Every effort should be made by the investigator to keep the subject in the study. Subjects who are withdrawn from investigational product will be replaced.

The primary reason for subject withdrawal from investigational product will be recorded in the CRF. Primary reasons for withdrawal will be categorized as:

Adverse event
Withdrew consent
Lost to follow-up
Lack of efficacy
Subject reached protocol-defined stopping criteria
Study closed/terminated
Investigator discretion

AS exacerbation may be a reason for withdrawal under lack of efficacy. Specific regard should be given to distinguish withdrawals due to an adverse event and lack of efficacy.

8.2 Screen Failures

A subject who has at least one study procedure performed signing a consent form, and is assigned a subject identifier but is not randomized is classified as a screen failure. The following information on subjects who are not randomized must be collected in the CRF:

Date screened

Subject identification number

Demography (race, age, and gender)

Reason subject failed Screening

Any Serious Adverse Events (SAEs) related to study procedures or concomitant medications, that occurred after signing the informed consent

A subject who is classified as a screen failure cannot be re-screened.

9.Adverse Event Reporting

Timely, accurate, and complete report and analysis of safety information are crucial for the protection of subjects, investigators, and the sponsor in the clinical trials.

9.1 Adverse Event

An adverse event is any untoward medical occurrence in a clinical study subject administered a pharmaceutical product. An adverse event does not necessarily have a causal relationship with the treatment. An adverse event can therefore be any unfavorable and unintended sign (including an abnormal finding), symptom, or disease temporally associated with the use of a medicinal (investigational) product, whether or not related to the medicinal (investigational) product (ICH GCP).

This includes any occurrence that is new in onset or aggravated in severity or frequency from the baseline condition, or abnormal results of diagnostic procedures, including laboratory test abnormalities.
Note: The sponsor collects adverse events starting with the first study-related procedure (not with the signing of the informed consent). The first study-related procedure might include discontinuation from or decrease in current therapy, a study-specific assessment or scale, or a study-specific procedure (e.g., laboratory test, X-ray, physical examination).

9.2 Serious Adverse Event

A serious adverse event is defined as any untoward medical occurrence that at any dose meets any of the following conditions:

 results in death

 is life-threatening (The subject was at risk of death at the time of the event. It does not

refer to an event that hypothetically might have caused death if it were more severe.)

requires inpatient hospitalization or prolongation of existing hospitalization

 results in persistent or significant disability/incapacity

 is a congenital anomaly/birth defect

Note: Important Medical Events: Medical and scientific judgment should be exercised in deciding whether expedited reporting is also appropriate in situations other than those listed above. For example, important medical events may not be immediately life threatening or result in death or hospitalization but may jeopardize the subject or may require intervention to prevent one of the outcomes listed in the definition above. Any adverse event is considered a serious adverse event if it is associated with clinical signs or symptoms judged by the investigator to have a significant clinical impact.

9.3 Unlisted (Unexpected) Adverse Event

An unlisted adverse event, the nature or severity of which is not consistent with the applicable product information. For an investigational product, the expectedness of an adverse event will be determined by whether or not it is listed in the Investigator's Brochure.

9.4 Associated With the Use of the Drug

Not related
An adverse event which is not related to the use of the drug.

Doubtful
An adverse event for which an alternative explanation is more likely, e.g., concomitant drug(s), concomitant disease(s), or the relationship in time suggests that a causal relationship is unlikely.

Possible
An adverse event which might be due to the use of the drug. An alternative explanation, e.g., concomitant drug(s), concomitant disease(s), is inconclusive. The relationship in time is reasonable; therefore, the causal relationship cannot be excluded.

Probable
An adverse event which might be due to the use of the drug. The relationship in time is suggestive (e.g., confirmed by de-challenge). An alternative explanation is less likely, e.g., concomitant drug(s), concomitant disease(s).

Very likely
An adverse event which is listed as a possible adverse reaction and cannot be reasonably explained by an alternative explanation, e.g. concomitant drug(s), concomitant disease(s). The relationship in time is very suggestive (e.g., it is confirmed by de-challenge and re- challenge).

10.Data Analysis/Statistical Methods

The data will be analyzed by using ANOVA, those do not meet normal distribution will be analyzed by Kruskal-Wallis test. Statistical primary comparisons for SPARCC scores will be made using a repeated measures mixed model with terms for treatment, center and the corresponding baseline values as the covariates. Chi-square will be used for the comparison of proportion rate in different groups.

Analysis is performed for the ITT population and PP population. ITT population is defined as all randomly assigned subjects who received at least 1 dose of test article and had efficacy evaluation after baseline. PP population is defined as the cases that completed all treatment, had no protocol violation and completed all evaluation of study. The efficacy and safety analysis will be performed in the ITT population. And the primary efficacy analysis is also performed for the per-protocol (PP) population

11.Ethics

11.1 Subject Information and Consent

Each subject (or a legally acceptable representative) must give written consent according to local requirements after the nature of the study has been fully explained. The consent form must be signed before performance of any study-related activity. The consent form that is used must be approved by both the sponsor and by the reviewing IEC/IRB. The informed consent should be in accordance with principles that originated in the Declaration of Helsinki, current ICH and GCP guidelines, applicable regulatory requirements, and sponsor policy.

Before entry into the study, the investigator or an authorized member of the investigational staff must explain to potential subjects or their legally acceptable representatives the aims, methods, reasonably anticipated benefits, and potential hazards of the study, and any discomfort it may entail. Subjects will be informed that their participation is voluntary and that they may withdraw consent to participate at any time. They will be informed that choosing not to participate will not affect the care the subject will receive for the treatment of his/her disease. Subjects will be told that alternative treatments are available if they refuse to take part and that such refusal will not prejudice future treatment. Finally, they will be told that the investigator will maintain a subject identification register for the purposes of long- term follow-up if needed and that their records may be accessed by competent authorities and authorized sponsor staff without violating the confidentiality of the subject, to the extent permitted by the applicable law(s) or regulations. By signing the informed consent form the subject or legally acceptable representative is authorizing such access.

The subject or legally acceptable representative will be given sufficient time to read the informed consent form and the opportunity to ask questions. After this explanation and before entry to the study, consent should be appropriately recorded by means of either the subject's or his/her legally acceptable representative's dated signature. After having obtained the consent, a copy of the informed consent form must be given to the subject.

If the subject or legally acceptable representative is unable to read or write, an impartial witness should be present for the entire informed consent process (which includes reading and explaining all written information) and personally date and sign the informed consent form after the oral consent of the subject or legally acceptable representative is obtained.

11.2. Investigator Responsibilities

The investigator is responsible for ensuring that the clinical study is performed in accordance with the protocol, current ICH guidelines on Good Clinical Practice (GCP), and applicable regulatory requirements.
GCP is an international ethical and scientific quality standard for designing, conducting, recording, and reporting studies that involve the participation of human subjects. Compliance with this standard provides public assurance that the rights, safety, and well beings of study subjects are protected, consistent with the principles that originated in the Declaration of Helsinki, and that the clinical study data are credible.

11.3 Independent Ethics Committee or Institutional Review Board (IEC/IRB)

Before the start of the study, the investigator (or sponsor where required) will provide the IEC/IRB with current and complete copies of the following documents:
final protocol and, if applicable, amendments sponsor-approved informed consent form (and any other written materials to be provided to the subjects)

Investigator’s Brochure (or equivalent information) and amendments sponsor-approved subject recruiting materials information on compensation for study-related injuries or payment to subjects for participation in the study, if applicable investigator’s curriculum vitae or equivalent information (unless not required, as documented by IEC/IRB) information regarding funding, name of the sponsor, institutional affiliations, other potential conflicts of interest, and incentives for subjects any other documents that the IEC/IRB requests to fulfill its obligation

This study will be undertaken only after IEC/IRB has given full approval of the final protocol, amendments (if any), the informed consent form, applicable recruiting materials, and subject compensation programs, and the sponsor has received a copy of this approval. This approval letter must be dated and must clearly identify the documents being approved. During the study the investigator (or sponsor where required) will send the following documents to the IEC/IRB for their review and approval, where appropriate:
protocol amendments revision(s) to informed consent form and any other written materials to be provided to subjects revisions to compensation for study-related injuries or payment to subjects for participation in the study, if applicable.
Investigator’s Brochure amendments or new edition(s) reports of adverse events that are serious, unlisted, and associated with the investigational drug new information that may affect adversely the safety of the subjects or the conduct of the study deviations from or changes to the protocol to eliminate immediate hazards to the subjects report of deaths of subjects under the investigator's care notification if a new investigator is responsible for the study at the site any other requirements of the IEC/IRB. For protocol amendments that increase subject risk, the amendment and applicable informed consent form revisions must be submitted promptly to the IEC/IRB for review and approval before implementation of the change(s).
At the end of the study, the investigator (or sponsor where required) will notify the IEC/IRB about the study completion.

11.4 Privacy of Personal Data

The collection and processing of personal data from subjects enrolled in this study will be limited to those data that are necessary to investigate the efficacy, safety, quality, and utility of the investigational product(s) used in this study.
These data must be collected and processed with adequate precautions to ensure confidentiality and compliance with applicable data privacy protection laws and regulations. The sponsor ensures that the personal data will be processed fairly and lawfully collected for specified, explicit, and legitimate purposes and not further processed in a way incompatible with these purposes adequate, relevant, and not excessive in relation to said purposes accurate and, where necessary, kept current.

Explicit consent for the processing of personal data will be obtained from the participating subject (or his/her legally acceptable representative) before collection of data. Such consent should also address the transfer of the data to other entities and to other countries.
The subject has the right to request through the investigator access to his/her personal data and the right to request rectification of any data that are not correct or complete. Reasonable steps should be taken to respond to such a request, taking into consideration the nature of the request, the conditions of the study, and the applicable laws and regulations.

Appropriate technical and organizational measures to protect the personal data against unauthorized disclosures or access, accidental or unlawful destruction, or accidental loss or alteration must be put in place. Sponsor personnel whose responsibilities require access to personal data agree to keep the identity of study subjects confidential.

11.5 Reporting of Safety Issues and Serious Breaches of the Protocol or ICH GCP

In the event of any prohibition or restriction imposed (clinical hold) by an applicable Competent Authority in any area of the World, or if the investigator is aware of any new information which might influence the evaluation of the benefits and risks of the investigational product, Pfizer should be informed immediately.

In addition, the investigator will inform Pfizer immediately of any urgent safety measures taken by the investigator to protect the study subjects against any immediate hazard, and of any serious breaches of this protocol or of ICH GCP that the investigator becomes aware of.
