# Supplementary material for: Etanercept/celecoxib on improving MRI inflammation of active ankylosing spondylitis: A multicenter, open-label, randomized clinical trial
Source: Front Immunol. 2022 Aug 26;13:967658. doi: 10.3389/fimmu.2022.967658 (PMC9458864; doi:10.3389/fimmu.2022.967658)
Supplement: Supplementary file 2 [file DataSheet_2.docx]

Table S1. Baseline characteristics of those completed and dropouts

|  | Completed  (n=133) | Dropout  (n=17) | *P*-value |
| --- | --- | --- | --- |
| Age (y) | 32.13±8.36 | 34.05±8.53 | 0.35 |
| Body mass index (kg/m2) | 22.87±3.78 | 21.20±4.05 | 0.08 |
| Disease duration (month) | 108.86±68.83 | 112.63±80.06 | 0.83 |
| BASDAI (0-10) | 5.20±2.19 | 5.38±2.35 | 0.75 |
| BASFI (0-10) | 3.09±2.34 | 2.95±2.14 | 0.81 |
| BASMI (0-10) | 2.24±2.17 | 2.84±2.24 | 0.26 |
| ASDAS-CRP | 3.61±0.86 | 3.55±0.97 | 0.80 |
| ASQoL | 8.21±4.92 | 7.42±4.63 | 0.51 |
| ESR | 27.48±22.77 | 26±25.59 | 0.79 |
| CRP | 22.76±21.63 | 22.79±33.53 | 0.99 |
| Syndesmophytes | 4.59±3.26 | 5±2.98 | 0.61 |
| mSASSS (0-36) | 11.95±6.44 | 13.21±6.79 | 0.43 |
| SIJ SPARCC (0-72) | 9.61±8.75 | 8.42±6.78 | 0.15 |
| Spine SPARCC (0-108) | 26.17±11.96 | 21.94±16.09 | 0.19 |
| SSS | 18.01±14.86 | 18.11±12.36 | 0.99 |

Values are mean (standard deviation) unless otherwise stated. Ttest was used for the comparisons.

BASDAI = Bath Ankylosing Spondylitis Disease Activity Index, BASFI = Bath Ankylosing Spondylitis Functional Index, BASMI= Bath Ankylosing Spondylitis Metroloty Index, ASDAS = Ankylosing Spondylitis Disease Activity Score, ASQoL= Ankylosing Spondylitis Quality of Life, ESR = Erythrocyte Sedimentation Rate, CRP = C-Reactive Protein, mSASSS = Modified Stoke Ankylosing Spondylitis Spine Score, SIJ = Sacroiliac joint, SPARCC = Spondyloarthritis Research Consortium of Canada, SSS = Sacroiliac Joint Structural Score.


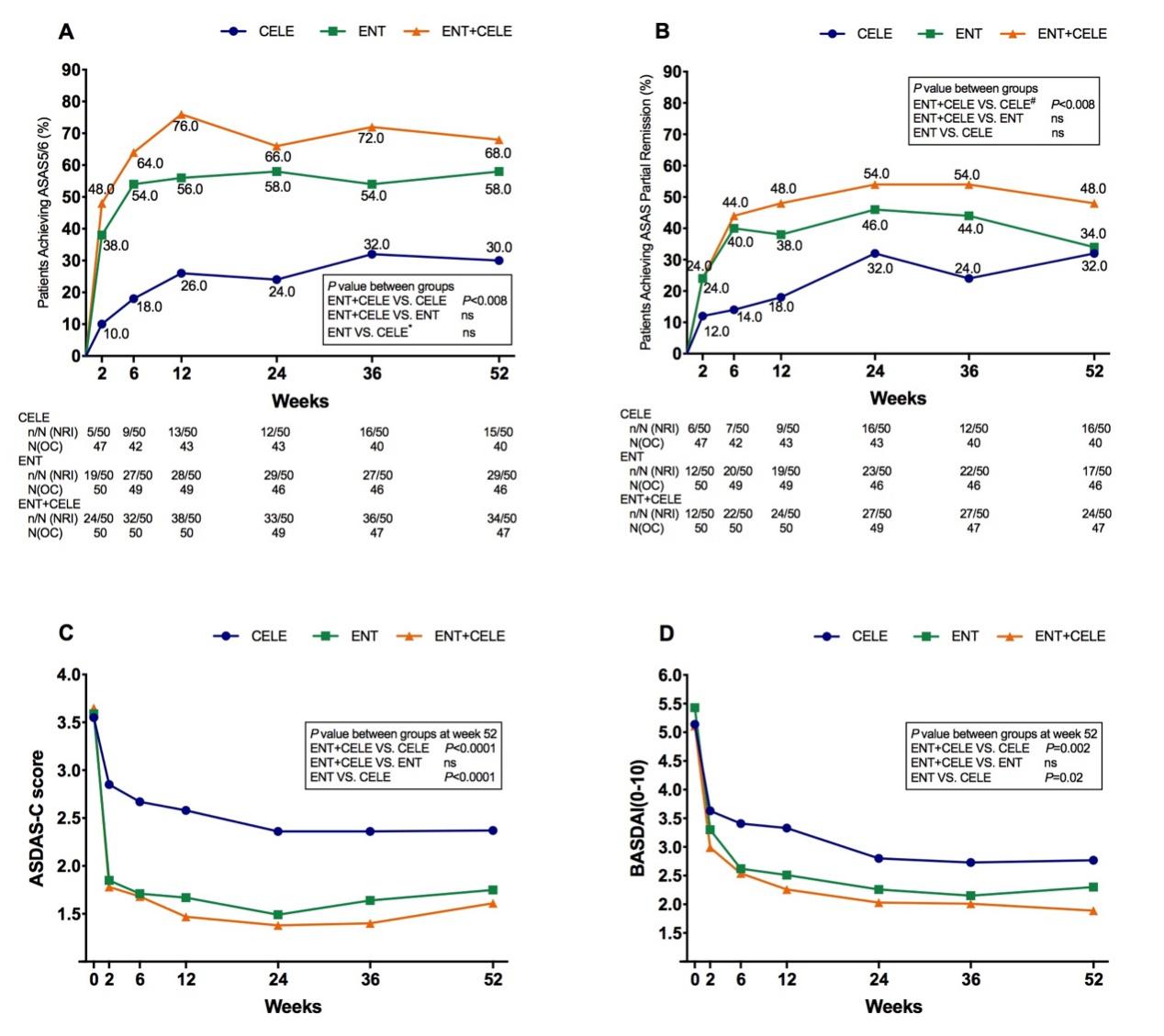


Figure S1. Proportion of patients achieving (A) ASAS5/6 response, (B) ASAS partial remission and change of (C) ASDAS-C score, (D) BASDAI score in three groups over 52 weeks. Population is modified intention to treat, non-responder imputation (NRI). The actual number of patients is shown as observed case (OC). P values for differences in results between groups at any time-points (ASAS5/6 and ASAS partial remission) are from Kruskal-Wallis test and adjusted p-value for significance is 0.008 in multiple comparisons between groups. Change of ASDAS-C and BASDAI score between-groups over week 52 are from mixed-effect model. ASAS, Assessment of SpondyloArthritis international Society; ASDAS, Ankylosing Spondylitis Disease Activity Score; BASDAI, Bath Ankylosing Spondylitis Disease Activity Index; ENT, etanercept; CELE, celecoxib; ns, non-significant.
